# Virtual Reality in Home Palliative Care: Brief Report on the Effect on Cancer-Related Symptomatology

**DOI:** 10.3389/fpsyg.2021.709154

**Published:** 2021-09-24

**Authors:** Serena Moscato, Vittoria Sichi, Andrea Giannelli, Pierpaolo Palumbo, Rita Ostan, Silvia Varani, Raffaella Pannuti, Lorenzo Chiari

**Affiliations:** ^1^Department of Electrical, Electronic, and Information Engineering “Guglielmo Marconi” – DEI, University of Bologna, Bologna, Italy; ^2^National Tumor Assistance (ANT) Foundation, Bologna, Italy; ^3^Health Sciences and Technologies – Interdepartmental Center for Industrial Research (CIRI-SDV), University of Bologna, Bologna, Italy

**Keywords:** anxiety, cancer, depression, digital health care, immersive technology, pain, palliative care, virtual reality

## Abstract

Virtual reality (VR) has been used as a complementary therapy for managing psychological and physical symptoms in cancer patients. In palliative care, the evidence about the use of VR is still inadequate. This study aims to assess the effect of an immersive VR-based intervention conducted at home on anxiety, depression, and pain over 4days and to evaluate the short-term effect of VR sessions on cancer-related symptomatology. Participants were advanced cancer patients assisted at home who were provided with a VR headset for 4days. On days one and four, anxiety and depression were measured by the Hospital Anxiety and Depression Scale (HADS) and pain by the Brief Pain Inventory (BPI). Before and after each VR session, symptoms were collected by the Edmonton Symptom Assessment Scale (ESAS). Participants wore a smart wristband measuring physiological signals associated with pain, anxiety, and depression. Fourteen patients (mean age 47.2±14.2years) were recruited. Anxiety, depression (HADS), and pain (BPI) did not change significantly between days one and four. However, the ESAS items related to pain, depression, anxiety, well-being, and shortness of breath collected immediately after the VR sessions showed a significant improvement (*p*<0.01). A progressive reduction in electrodermal activity has been observed comparing the recordings before, during, and after the VR sessions, although these changes were not statistically significant. This brief research report supports the idea that VR could represent a suitable complementary tool for psychological treatment in advanced cancer patients assisted at home.

## Introduction

Cancer significantly interferes in all aspects of daily life, from family and personal relationships to business and financial areas (Lentz et al., 2019; Schouten et al., 2019). In addition to compromising the patient’s physical health, the oncological disease often causes psychological discomfort characterized by anxious and depressive symptoms that may be clinically significant (Pitman et al., 2018; Niedzwiedz et al., 2019; Mausbach et al., 2020). Many cancer patients experience severe levels of psychological discomfort, with a percentage varying from 10–15% to 20–40% (Mitchell et al., 2011; Shim et al., 2018). The average prevalence of mood disorders ranges from 29% in palliative care (PC) settings to 38% in cancer and hematological contexts (Jacobsen et al., 2005). In addition to the emotional distress related to the anguish of facing death, the advanced stages of the disease often imply a progressive loss of functional autonomy (Dong et al., 2016). This puts the patient in a status of deprivation and forced isolation from relational and social aspects that were hitherto in their lives. This psychological discomfort naturally adds to the burden of the physical symptoms caused by the disease and/or invasive therapies, above all pain.

In the last years, technological innovation has allowed new solutions to meet old and emerging needs in the healthcare field. One of these is represented by virtual reality (VR), a non-invasive simulation technology that allows the user to be immersed in a multisensory experience. VR technologies can emotionally involve the patients, inducing a positive mood and allowing them to feel like a part of the virtual environment (Malloy and Milling, 2010; Pourmand et al., 2018; Ahmadpour et al., 2019). The distraction effect is recognized to be the primary principle underlying the effectiveness of VR, as it is capable of diverting patient’s attention from his or her current clinical condition (Schneider et al., 2004; Malloy and Milling, 2010; Wiederhold et al., 2014; Bani Mohammad and Ahmad, 2019). The type of content also plays a key role: Natural contents have been shown to induce relaxation and restore work productivity, based on the Attention Restoration Theory (Kaplan, 1995; Berto, 2005; Anderson et al., 2017), while aquatic scenes with views and the sound of water seem to be more appreciated than scenes without water (White et al., 2010; Anderson et al., 2017). Additionally, according to Berto (2005), interactive contents that demand higher attentional capacity could be even more effective in their distraction-based effect than contents that only require passive observation (Wiederhold and Wiederhold, 2007; Shahrbanian et al., 2012; Wender et al., 2019). From a practical point of view, immersive VR-based interventions are a convenient solution, as they do not require complicated training to be used and can be employed in case of low mobility conditions.

VR has been used as an adjuvant treatment in various clinical conditions to relieve pain, anxiety, and depression (Dascal et al., 2017; Fodor et al., 2018), during invasive procedures (Hoffman et al., 2011; Shetty et al., 2019; Brown and Foronda, 2020), for chronic pain management (Wiederhold et al., 2014; Pekyavas and Ergun, 2017), and in rehabilitation settings (Shahrbanian et al., 2012; Lee et al., 2015). It has been extensively employed in promoting cancer patients’ psychological well-being (Chirico et al., 2016; Zeng et al., 2019; Ioannou et al., 2020). Previous studies assessed the effects of VR in relieving cancer-related symptomatology (i.e., sadness, stress, and anxiety) mainly in clinical settings (Li et al., 2011; Baños et al., 2013; Mosadeghi et al., 2016; Bani Mohammad and Ahmad, 2019) and in patients undergoing painful procedures (Gershon et al., 2004; Hoffman et al., 2014), alleviating anxiety and improving mood states. Studies in PC have proven the acceptability of VR interventions (Oyama, 1997; Brungardt et al., 2020; Weingarten et al., 2020) and their effects in reducing short-term symptoms (i.e., pain, drowsiness, lack of appetite, shortness of breath, depression, anxiety, and well-being; Niki et al., 2019; Johnson et al., 2020).

The evidence about the use of immersive VR in a home-PC program is still insufficient. To fill this gap, the National Tumor Assistance Foundation (ANT), a non-profit organization working in Italy in the field of home-PC and pain management, conducted a pilot study to test the feasibility of VR interventions for cancer patients assisted at home (Varani et al., 2018). To the best of our knowledge, this was the first attempt to introduce VR interventions in home-PC settings, obtaining promising results: Most participants (73%) declared that they would be willing to keep a VR headset at home; patients showed a 20% decrease in anxiety and depression and a significant reduction in perceived pain and fatigue symptoms. As a further outcome, ANT collected patients’ preferences as the output of a focus group and semi-structured interviews: All patients declared to prefer natural and relaxing scenarios, and 36% expressed a preference for interactive videos, while 33% wanted to see videos as simple observers (Sichi et al., 2019).

After these encouraging results, ANT introduced the VR headset with interactive and non-interactive contents in its home care practice. Starting from the hypothesis that VR can be used as an effective adjuvant intervention in home-PC to improve anxiety, depression, pain, and other symptoms in advanced cancer patients, this brief report describes the results obtained during the first 18months of this ongoing experience. This pre-post single-arm study aims to reach three objectives:

- To assess the effect of a VR-based intervention at reducing anxiety, depression, and pain over 4 days;
- To evaluate the short-term effect of VR sessions on cancer-related symptomatology and physiological signals;
- To explore whether the two contents (i.e., interactive vs. non-interactive) can have a different effect on the cancer-related symptomatology.

## Materials and Methods

### Participants

Participants were advanced cancer patients assisted within the home-PC program of ANT Foundation in the metropolitan area of Bologna (Italy). We excluded patients who were under 18 and over 70, with visual and hearing impairment, unable to understand the informed consent, to answer the questionnaires due to cognitive impairment, to understand the Italian language, with a diagnosis of epilepsy, dementia, or other neurological disorders, brain cancer or brain metastases, or heart attack in the last year. Each participant signed written informed consent for intervention and data collection before starting the study. Data collection lasted from May 2019 to July 2020. To verify the patients’ eligibility, ANT psychologists discussed with the ANT physician, evaluating whether the patient respected all inclusion criteria. After that, the psychologists contacted the patient to propose and explain the study design.

### Measures

#### Socio-Demographic and Clinical Data

We collected data on gender, civil status, cohabitation, occupation, education, and confidence with technology by a questionnaire. Cancer diagnosis, Karnofsky Performance Score (KPS; Karnofsky and Burchenal, 1949), extension of pathology, and therapies were detailed from the patient’s medical records.

#### Hospital Anxiety and Depression Scale

Zigmond and Snaith (1983). We used the Italian validated version of Hospital Anxiety and Depression Scale (HADS; Costantini et al., 1999). HADS is a simple and brief scale consisting of 14 items exploring both anxiety and depression symptoms. The main characteristic of the scale is that it excludes the somatic symptomatology from the evaluation. The scale instead focuses – especially for depression – on the reduction in hedonic capacity, considering it among the most sensitive indicators in diagnosing this disorder (Snaith, 2003).

#### Brief Pain Inventory

The validated Italian version of Brief Pain Inventory (BPI; Caraceni et al., 1996; Bonezzi et al., 2002) is an easy-to-use questionnaire assessing the intensity of pain, the interference of pain with the patient’s life, pain relief, pain quality, and patient perception of the cause of pain.

#### Edmonton Symptom Assessment Scale

Chang et al. (2000); Hui and Bruera (2017). The Italian validated version of Edmonton Symptom Assessment Scale (ESAS; Moro et al., 2006) is a simple and reliable multi-item instrument developed to rate the intensity of nine common symptoms experienced by cancer patients (pain, tiredness, nausea, depression, anxiety, drowsiness, appetite, well-being, and shortness of breath).

#### Physiological Parameters

We used a smart wearable wristband in order to evaluate the psychophysiological effects of the VR, as also reported in other studies (Anderson et al., 2017; Ishaque et al., 2020). We selected electrodermal activity (EDA), heart rate (HR), and skin temperature (SKT) as representative of the activation of the autonomic nervous system (Hui and Sherratt, 2018). These physiological signals and the related features have been extensively used in the literature in the field of emotion recognition (Egger et al., 2019; Li et al., 2021). Specifically, they have been used in association with stress and pain (Campbell et al., 2019; Chen et al., 2021; Chesnut et al., 2021) and to evaluate depressive and anxious symptoms (Sarchiapone et al., 2018; Chesnut et al., 2021). We hypothesized a reduction in the physiological signals’ amplitude due to the VR use, reflecting a more relaxed state of the patient. Physiological parameters were derived from recordings of the Empatica E4 wristband, a CE medical wearable device already tested for its reliability (Sabeti et al., 2019; Schuurmans et al., 2020). We also quantified a motor activity level, that is, the Activity Index (AI), on one-minute time windows by processing the accelerometer signal (Lin et al., 2018). The quality of the EDA recordings was assessed with the algorithm reported in Taylor et al. (2015). This algorithm automatically detects corrupted segments on a 5-s time window basis and replaces them with a linear interpolation between the last and the following good quality segment. Empatica proprietary algorithm automatically provides reliable interbeat interval (IBI) from photoplethysmographic signal, from which the heart rate (HR) is then derived. We did not assess quality for SKT and AI as they are not affected by movement artifacts. We averaged each of these four signals (EDA, HR, SKT, and AI) during each session and on 10-min windows before and after each session.

### Interventions

This study has been designed as a pre-post single-arm study. The intervention was based on the supply of a VR headset with customized contents. The headset was Mirage Solo VR (LENOVO S.r.l.) – including a remote controller – supplied at the home of the participant for 4days.

The ANT psychologist suggested using the headset in moments of psychophysical discomfort (e.g., pain and growing anxiety). We did not set a minimum or a maximum on usage time nor on the number of sessions, in order to not force the use of the VR headset. Participants were also instructed to take off the VR headset immediately if they had any side effect and to promptly inform the investigator for having support. Possible side effects reported in the VR headset user manual (Lenovo, 2018) are dizziness, motion sickness, nausea, disorientation, convulsion, and altered vision.

The VR headset was provided with both interactive and non-interactive contents specifically developed for ANT patients by Immerxive Srl, (2016), on the basis of the pilot study outcomes (Varani et al., 2018; Sichi et al., 2019) and under the supervision of ANT psychologists. Specifically, the non-interactive contents consisted of immersive 360° videos with different natural and relaxing scenarios, such as a seascape, a park, a waterfall, the London Bridge, and a mountain landscape. The interactive content consisted of a basic skill game called “Yuma’s World”: At each level, the user, surrounded by a calm underwater environment, had to reproduce with the controller a displayed “Kanji,” that is, a Japanese ideogram that represents concepts like friendship, courage, and strength. Snapshots of these interactive and non-interactive contents are provided in Supplementary Figure 1.

**Figure 1:**
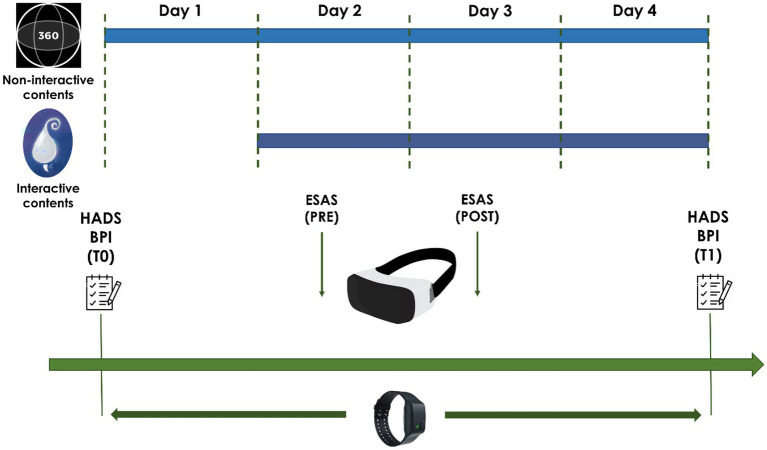
Graphical representation of the VR-based intervention.

Patients received the VR headset and kept it at home for 4days. On the first day (T0), an ANT psychologist described the study to participants, who then signed the informed consent form. The psychologist asked them to answer the socio-demographic form, the HADS (Zigmond and Snaith, 1983)^,^ and the BPI (Caraceni et al., 1996; Bonezzi et al., 2002). The clinical data form was filled in by the psychologist based on the medical records. Before and after each use of the VR headset, the patient completed a digital version of the ESAS (Bruera et al., 1991) directly through the VR headset. At T0, the participants could only watch the non-interactive contents in order to familiarize themselves with the VR. From the second day on, participants received a password to unlock the interactive content. We assumed that this strategy could produce a surprise effect in the patients, encouraging them the use of VR. From day 2, participants are free to choose the type of content (interactive vs. non-interactive) whenever they use the VR headset. Participants were also asked to wear the E4 wristband (Empatica Srl, 2014) to record physiological signals continuously from T0 to T1, except when showering. At T1, patients completed HADS and the BPI again, and the ANT psychologist took back the VR headset and the E4 wristband. A graphical depiction of the study is provided in Figure 1.

### Statistical Analyses

After checking the non-normality with a Kolmogorov-Smirnov test, changes in anxiety, depression (HADS), and pain (BPI) between T0 and T1 were investigated with a Wilcoxon signed-rank test. To evaluate the short-term effects of VR on ESAS items and physiological signals, we used linear mixed effect models (LMMs; (Goldstein et al., 2002), taking into account multiple measures for each subject. In particular, we fitted 13 LMMs (9 for the ESAS items and 4 for the physiological parameters), where: the outcome variable was each ESAS item or physiological parameter; the fixed effect was a “pre-post session” variable for the ESAS items and a “pre-during-post session” variable for the physiological parameters; and patient and VR session were the random effects. To assess the possible impact of the different multimedia content (i.e., interactive vs. non-interactive), we used LMMs, where: the difference between the ESAS scores rated after and before the VR session was set as the outcome variable; a variable specifying the interactive and non-interactive content was included as a fixed effect, and the patient was modeled as a random effect. We drew inference on the LMMs’ fixed effects (differences between pre-(during)-post and types of multimedia contents) with a chi-square test for nested models, comparing the likelihood of each LMM with and without the fixed effect. In order to evaluate whether the usage time had an effect on the improvement of cancer-related symptomatology, we carried out a correlation analysis. For HADS and BPI scores, we assessed the correlation between the total usage time for each participant and the post-pre difference. For ESAS scores, we assessed the correlation between the usage time for the single VR session and the post-pre difference.

Statistical analyses for HADS, BPI, and ESAS questionnaires were run on R (R Core Team, 2019), using *wilcox.test* function and the *lme4* package (Bates et al., 2015) to fit LMMs. The processing and the analysis of the physiological signals were performed in Matlab (Matlab, 2020), using the *fitlme* built-in function to fit LMMs.

## Results

### Study Population

Participants were recruited from Bologna metropolitan area. The study was proposed to 25 ANT patients. Eleven patients refused to participate primarily due to family or disease-related reasons. The final sample consists of 14 participants (3 men and 11 women, mean age 47.2±14.2years; Table 1). Most of the patients were married or cohabitant (71.4%), employed (50.0%) and had a high school diploma educational level (57.1%). All the participants declared to be confident with technology like TV/cinema and most of them with PC/smartphone (85.7%). Only two participants (14.3%) stated to be familiar with videogames or 3D movies and only one of them (7.1%) to be confident with VR headsets.

**Table 1:** Socio-demographic and clinical data of the participants.

**Sample size**	*N*=14
**Age, mean (S.D.)**	47.2 (±14.17)
**Gender**	
Men, n (%)	3 (21.4%)
Women, n (%)	11 (78.6%)
**Civil status**	
Married/Cohabitant, n (%)	10 (71.4%)
Unmarried, n (%)	3 (21.4%)
Widowed, n (%)	1 (7.2%)
**Cohabitation with**	
Partner, n (%)	5 (35.7%)
Partner and offspring, n (%)	4 (28.6%)
Parents, n (%)	2 (14.3%)
Alone, n (%)	2 (14.3%)
Other, n (%)	1 (7.1%)
**Occupation**	
Employed, n (%)	7 (50.0%)
Retiree/off work, n (%)	4 (28.6%)
Student, n (%)	2 (14.3%)
Unemployed, n (%)	1 (7.1%)
**Education**	
High school diploma, n (%)	8 (57.1%)
Degree, n (%)	6 (42.9%)
**Confidence with this technology**	
TV/cinema, n (%)	14 (100.0%)
PC/smartphone, n (%)	12 (85.7%)
Videogames, n (%)	2 (14.3%)
3D film in TV/cinema, n (%)	2 (14.3%)
Virtual Reality headset, n (%)	1 (7.1%)
Karnofsky Performance Score**, mean (S.D.)**	77.1 (±17.3)
**Cancer diagnosis**	
Gastrointestinal, n (%)	4 (28.6%)
Genital tract, n (%)	2 (14.3%)
Hematological, n (%)	2 (14.3%)
Breast, n (%)	2 (14.3%)
Urinary, n (%)	2 (14.3%)
Bone and soft tissues, n (%)	1 (7.1%)
Endocrine, n (%)	1 (7.1%)
**Extension of pathology**	
Localized, n (%)	7 (50.0%)
Metastatic, n (%)	7 (50.0%)
**Drugs**	
None, n (%)	5 (35.7%)
Pain medication, n (%)	7 (50.0%)
Pain medicatio n and antidepressant, n (%)	1 (7.1%)
Pain medication, anxiolytic, antidepressant, n (%)	1 (7.1%)

Clinical data showed that participants had a mean KPS of 77.1 (±17.3). The most common primary site of the tumor was the gastrointestinal tract (28.6%); seven patients (50.0%) suffered from localized cancer, and seven patients (50.0%) suffered from metastatic cancer. Five participants (35.7%) did not assume drugs, one participant (7.1%) took pain medication together with antidepressants, one participant (7.1%) took pain medication together with anxiolytics and antidepressants, and seven participants (50.0%) took only pain medication (Table 1).

No negative side effect during or after the use of VR was reported.

A total of 805h and 31min of physiological signals were collected during the 4-day observation period. During the VR sessions, 10h and 39min were recorded, during which EDA and IBI signals were classified as high quality for 88% and 36% of the time, respectively (see Supplementary Table 1). Supplementary Figure 2 shows an example of physiological recording, with a zoom-in of a VR session.

### Use of VR Headset

A total of 73 VR sessions from 14 participants were collected (Table 2). The total usage time was 13h and 2min, with an average of 55min per participant. Each patient used the VR at least one time, with a minimum of 3min (for more details, see Supplementary Table 2). Non-interactive contents were enjoyed more than interactive contents in terms of the number of sessions, although the total usage time of the VR experience was quite similar between the two types of content (non-interactive contents: 6h and 58min; interactive contents: 6h and 12min). Most of the VR sessions were concentrated in the first 2days, and the VR use diminished toward the end of the observation period.

**Table 2:** Usage time of VR use from all participants.

		**No. VR sessions**	**Usage time** (hh:mm)	**Non-interactive**	**Usage time** (hh:mm)	**Interactive**	**Usage time** (hh:mm)
Day 1	**Tot**	28	04:07	28	04:04	0	00:00
	**Mean**	2.00 (0.96)	00:17 (00:17)	2.00 (0.96)	00:17 (00:17)	0	00:00 (00:00)
Day 2	**Tot**	22	04:48	10	01:56	12	03:07
	**Mean**	1.57 (1.45)	00:20 (00:23)	0.71 (0.73)	00:08 (00:14)	0.86 (0.86)	00:13 (00:12)
Day 3	**Tot**	14	02:35	4	00:32	10	02:03
	**Mean**	1 (0.78)	00:11 (00:07)	0.29 (0.61)	00:02 (00:04)	0.71 (0.47)	00:08 (00:06)
Day 4	**Tot**	9	01:25	3	00:28	6	00:57
	**Mean**	0.64 (1.28)	00:06 (00:13)	0.21 (0.58)	00:02 (00:07)	0.43 (0.94)	00:04 (00:09)
Total	**Tot**	73	13:02	45	06:58	28	06:12
	**Mean**	5.21 (2.99)	00:55 (00:49)	3.21 (1.85)	00:29 (00:38)	2.00 (1.71)	00:26 (00:21)

For each day, in the first column, the total number of VR sessions, the mean number of VR sessions for each participant, and the related usage time are shown as mean (standard deviation). The same information is given for each type of multimedia content (non-interactive and interactive). In the last row, the overall descriptive statistics for the entire usage time of the experiment from all participants are reported.

### Outcomes

The results of the T0-T1 and immediate effects of VR are shown in Table 3.

**Table 3:** Results from the statistical analysis for Hospital Anxiety and Depression Scale (HADS), Brief Pain Inventory (BPI), Edmonton Symptom Assessment Scale (ESAS), and physiological parameters.

**HADS score**	**T0**		**T1**	**r**	**p value** ^**a**^
Anxiety	7.0 (5.79–11.78)		8.0 (4.97–11.03)	0.69	0.24
Depression	5.5 (2.52–6.33)		6.5 (2.73–6.69)	0.35	0.49
**BPI score**	**T0**		**T1**	**r**	**p value** ^**a**^
Worst pain	1.5 (0.73–3.98)		1.0 (0.69–4.45)	0.27	0.56
Mildest pain	0.5 (0.21–2.64)		1.0 (0.45–2.14)	0.49	0.64
Mean pain	1 (0.38–3.18)		1.5 (0.56–2.43)	0	0.25
Current pain	0 (0.21–1.78)		0.5 (0.27–2.01)	0.10	0.75
**ESAS score**	**Pre VR**		**Post VR**	**Δ** (Post - Pre)	**p value** ^**b**^
Pain	1.06 (0.25)		0.66 (0.10)	−37.73%	**<0.01**
Tiredness	4.04 (0.71)		3.91 (0.23)	−3.22%	0.56
Nausea	0.31 (0.16)		0.31 (0.07)	0%	1.00
Depression	1.83 (0.43)		1.28 (0.13)	−30.05%	**<0.01**
Anxiety	2.20 (0.40)		1.18 (0.16)	−46.36%	**<0.01**
Drowsiness	1.63 (0.40)		1.34 (0.15)	−17.18%	0.06
Appetite	3.89 (0.57)		3.63 (0.20)	−6.68%	0.19
Well-being	3.87 (0.39)		2.85 (0.21)	−26.36%	**<0.01**
Shortness of breath	2.01 (0.44)		1.28 (0.20)	−36.32%	**<0.01**
**Physiological**	**Pre VR**	**During VR**	**Post VR**	**Δ (Post - Pre)**	**p value** ^**b**^
**parameters**					
EDA	1.15 (0.36)	0.86 (0.33)	0.60 (0.33)	−30.43%	0.25
HR	85.11 (1.96)	83.58 (1.10)	85.56 (1.10)	+0.53%	0.17
SKT	33.49 (0.31)	33.24 (0.31)	33.73 (0.31)	+0.71%	0.07
AI	1.25 (0.36)	1.04 (0.13)	1.35 (0.13)	+8.00%	0.29

a Wilcoxon signed-rank test.

b chi-square test.

HADS and BPI: Data are shown as the median (95% C.I.) of both tests collected at T0 and T1. ESAS: Data are shown as the mean (S.E.M.) of all the VR sessions for test item. *r*=rank correlation. Δ=relative difference.

Anxiety, depression (HADS), and pain (BPI) levels did not change significantly between T0 and T1.

The nine LMMs of the ESAS items showed a significant improvement in pain, depression, anxiety, well-being, and shortness of breath immediately after using the VR. The four LMMs of the physiological parameters (EDA, HR, SKT, and AI) showed no significant changes before, during, and after the VR sessions. Albeit not statistically significant, the EDA showed a progressive decrease during the three phases, while the HR, SKT, and AI had a reduction during the VR sessions compared to the 10-min time windows before and after the intervention.

From the correlation analysis between total usage time for each participant and post-pre differences in HADS and BPI scores, we found a significant positive correlation for the “mean pain” BPI item (*r*=0.63, *p*<0.05). On the other hand, from the correlation analysis between usage time of the single VR session and post-pre differences in ESAS scores, we found significant negative correlations for anxiety (*r*=−0.27, *p*<0.05), worst feeling of well-being (*r*=−0.25, *p*<0.05), and shortness of breath (*r*=−0.28, *p*<0.05).

The comparison of the effect on ESAS symptoms between non-interactive and interactive contents revealed no significant difference (*p*>0.05 for all the items, Supplementary Table 3).

## Discussion

The supply of a VR headset to advanced cancer patients at home did not determine a significant improvement on pain (BPI), anxiety, or depression (HADS) over a four-day period. Nonetheless, VR sessions showed an immediate effect in relieving patient-reported symptomatology (ESAS), and no significant change in physiological parameters (HR, EDA, AI, and SKT) could be appreciated. No differential effect was found between interactive and non-interactive VR contents. To our knowledge, this research and the previous pilot study (Varani et al., 2018) are among the first studies investigating the effects of immersive VR on advanced cancer patients assisted at home. The data of this brief report were collected by the psychologists of a PC team during an 18-month period of home care practice.

In this setting, the VR intervention was appreciated by participants, and no one reported adverse side effects caused by its use. In agreement with these results, several studies confirmed user satisfaction, comfort, and feasibility of VR-based psychological interventions on patients with severe medical conditions (Baños et al., 2013), in PC patients (Johnson et al., 2020), and in older adults with an impaired sensory capacity, mobility, and/or cognition (Appel et al., 2020).

As a further step, this pre-post study assessed the degree of voluntary use of the VR headset at home over multiple days. All patients used at least once the VR device during the intervention, though the VR usage time, and the number of sessions decreased over the days. This could be due to the reduction in “the element of surprise” given by the novelty of the VR experience. Therefore, the inclusion of a variety of multimedia contents could be crucial to maintain the patients involved in the VR treatment for a longer period. To encourage an increased use of the device, ANT Foundation is working to implement new and more enjoyable VR contents.

In the pilot study, Varani et al. (2018) found a positive effect of VR on HADS. Similarly, Chan and colleagues found a significant improvement on HADS and State Trait Anxiety Inventory immediately after a 10-min VR session in women undergoing gynecological surgery (Chan et al., 2020). These different findings could be explained by the small sample size and the different design of those interventions. In particular, in both Varani et al. (2018) and Chan et al. (2020), patients filled the questionnaires immediately after the last VR session. In the present report, anxiety and depression were evaluated after 4days of VR utilization. Also, the results on BPI of this study confirm findings of the literature that the benefits of VR utilization on the participant’s pain do not persist long after post-exposure (Garrett et al., 2017).

The absence of long-term effects assessed by HADS and BPI could be also due to other factors. For instance, as shown by Supplementary Table 2, only few patients used the VR headset along the 4days, thus limiting the VR effects. Moreover, it could be possible that HADS and BPI outcomes can be influenced by uncontrolled or unreported adverse events over the 4days not related to the VR treatment.

The analysis of ESAS immediately before and after each VR session showed a significant reduction in pain, depression, anxiety, shortness of breath, and an improved well-being. These findings are in agreement with those of Niki and colleagues, demonstrating that a one-time VR intervention is sufficient to significantly reduce pain, tiredness, drowsiness, shortness of breath, depression, and anxiety measured by ESAS in a group of terminal cancer patients (Niki et al., 2019). Another study involving a small group of hospice patients undergoing a 30-min VR intervention described a trend for a reduction in ESAS scores of pain, tiredness, drowsiness, depression, and anxiety (Johnson et al., 2020). The improvements of the symptomatology observed immediately after using the VR device could be ascribed to the distraction effect of the VR technology. Other researchers suggested that the deep form of distraction produced by VR experiences is the primary mechanism by which pain is attenuated (Schneider et al., 2004; Malloy and Milling, 2010; Wiederhold et al., 2014; Bani Mohammad and Ahmad, 2019). Considering the fragile clinical conditions and short life expectancy of PC patients, we believe that getting relief from the symptoms, although only for a short period, represents a remarkable achievement.

Relevant insights on short-term effects can also be derived from physiological signals. Such these signals have already been used in clinical research to assess the subjects’ arousal in response to different events. Moreover, physiological signals are often employed as a complementary tool in oncological pain assessment (Masel et al., 2016; Delmotte et al., 2018). The progressive EDA decrease that we observed before, during, and after the intervention agrees with Anderson and colleagues, who found a reduction in the EDA signal in subjects experiencing VR natural scenes (Anderson et al., 2017). EDA decrease can be seen as an effect of a progressive de-activation of the sympathetic branch of the autonomic nervous system (Hui and Sherratt, 2018). The lack of statistical significance could be due to numerous reasons. For example, we extracted the mean of each signal as the only representative feature and on a fixed-length time window. As a future study, we suggest carrying out a more in-depth analysis with a larger sample size on other features detected across longer time windows.

We investigated whether the usage time could be related to an improvement in cancer-related symptomatology. For the long-term effects (i.e., HADS and BPI), we found a significant positive correlation with the “mean pain” BPI item. Since participants were asked to use the VR headset in moments of psychophysical discomfort, this positive correlation could mean that those patients with a worsening of pain used more the VR headset. On the other hand, for the short-term effects (i.e., ESAS), we found significant negative correlations between usage time of the single VR session, anxiety, and other related symptoms (i.e., shortness of breath and well-being). This could represent an effective improvement provided by a longer VR usage time.

No differential effect was found between interactive and non-interactive VR contents. Since interactive contents require more attentive resources, they are expected to have a more substantial distractive effect (Wiederhold and Wiederhold, 2007; Shahrbanian et al., 2012) and, in turn, to more effectively relieve cancer-related symptomatology. It is worth noting that the number of interactive VR sessions is far less than the number of non-interactive ones (28 vs. 45), and the VR headset was endowed with a single interactive content against five non-interactive ones. Further studies introducing additional interactive contents could lead to more conclusive results.

This study presents some limitations. According to the aims and the design of the study, patients were instructed to freely use the VR headset at home. This can introduce many factors that cannot be controlled, such as the number of VR usages and the context in which the patient used the VR. Other equally important aspects are the small sample size and the absence of a control group. Eventually, there may be several confounding effects. For example, patients who feel worse could use more VR. This can influence the results and cannot be corrected due to the lack of a control group. All these factors limit the strength of the conclusion drawn about whether any effects of the intervention can be ascribed solely to the VR. Despite these limitations, this is a study on a real-world population based on a daily home care practice. We tested the use of VR in real-world condition, and from this pre-post study, we were able to detect interesting aspects about the use of the VR in home palliative care settings that will be deepened in a next randomized controlled trial.

This brief research report supports that immersive audiovisual technology, such as VR, could represent a suitable tool to integrate the standard psychological support for patients with advanced cancer to improve their well-being with an immediate effect on their symptomatology. For the first time, this intervention brought the VR device to the PC patients’ homes. The participants could benefit from this technology freely and use it in moments of worst psychophysical discomfort. Indeed, this approach is highly innovative, and it is worthy of deeper investigations in combination with other distraction and/or relaxation techniques in future larger-scale studies.

## Data Availability Statement

The raw data supporting the conclusions of this article will be made available by the authors, without undue reservation.

## Ethics Statement

The studies involving human participants were reviewed and approved by the Ethical Committee of Area Vasta Emilia Centro (Bologna, Italy; approval n° 542-2019-OSS-AUSLBO). The patients/participants provided their written informed consent to participate in this study.

## Author Contributions

SM: conceptualization, investigation, methodology, data analysis, and writing original draft. VS: conceptualization, investigation, methodology, data collection, and writing original draft. AG: investigation, data collection, and writing original draft. PP: methodology, data analysis, and writing review and editing. RO: supervision, project administration, and writing review and editing. SV: conceptualization, supervision, and project administration. RP: supervision and project administration. LC: conceptualization, supervision, and project administration. All authors contributed to the article and approved the submitted version.

## Conflict of Interest

The authors declare that the research was conducted in the absence of any commercial or financial relationships that could be construed as a potential conflict of interest.

## Publisher’s Note

All claims expressed in this article are solely those of the authors and do not necessarily represent those of their affiliated organizations, or those of the publisher, the editors and the reviewers. Any product that may be evaluated in this article, or claim that may be made by its manufacturer, is not guaranteed or endorsed by the publisher.
