# Supplementary material for: Virtual Reality in Home Palliative Care: Brief Report on the Effect on Cancer-Related Symptomatology
Source: Front Psychol. 2021 Sep 24;12:709154. doi: 10.3389/fpsyg.2021.709154 (PMC8497744; doi:10.3389/fpsyg.2021.709154)
Supplement: Supplementary file 1 [file Data_Sheet_1.docx]

**Supplementary Table 1 –** Physiological recordings and EDA and IBI quality

|  |  | **No. VR sessions** | **Duration**  (hh:mm) | **High quality EDA**  (hh:mm) | **High quality IBI**  (hh:mm) |
| --- | --- | --- | --- | --- | --- |
| Day 1 | **Tot** | 12 | 02:31 | 02:09 | 00:46 |
|  | **Mean** | 0.86 (1.10) | 00:10 (00:17) | 00:09 (00:15) | 00:03 (00:06) |
| Day 2 | **Tot** | 22 | 04:43 | 04:28 | 01:46 |
|  | **Mean** | 1.57 (1.45) | 00:20 (00:23) | 00:19 (00:23) | 00:07 (00:09) |
| Day 3 | **Tot** | 14 | 02:26 | 01:40 | 00:45 |
|  | **Mean** | 1 (0.78) | 00:10 (00:08) | 00:07 (00:07) | 00:03 (00:04) |
| Day 4 | **Tot** | 9 | 01:15 | 01:10 | 00:31 |
|  | **Mean** | 0.64 (1.28) | 00:05 (00:13) | 00:05 (00:12) | 00:02 (00:06) |
| Total | **Tot** | 73 | 10:39 | 09:22 | 03:51 |
|  | **Mean** | 5.21 (2.99) | 00:45 (00:50) | 00:40 (00:49) | 00:16 (00:21) |

**Supplementary Table 2** – VR usage time for each patient

| **# P** | **Complete VR sessions** | | | | | | **Day 1** | | | | | | **Day 2** | | | | | | **Day 3** | | | | | | **Day 4** | | | | | |
| --- | --- | --- | --- | --- | --- | --- | --- | --- | --- | --- | --- | --- | --- | --- | --- | --- | --- | --- | --- | --- | --- | --- | --- | --- | --- | --- | --- | --- | --- | --- |
|  | **#S** | **UT** | **NI** | **UT** | **I** | **UT** | **#S** | **UT** | **NI** | **UT** | **I** | **UT** | **#S** | **UT** | **NI** | **UT** | **I** | **UT** | **#S** | **UT** | **NI** | **UT** | **I** | **UT** | **#S** | **UT** | **NI** | **UT** | **I** | **UT** |
| 1 | 3 | 0:20 | 2 | 0:11 | 1 | 0:09 | 2 | 0:13 | 2 | 0:11 | 0 | 0:00 | 0 | 0:00 | 0 | 0:00 | 0 | 0:00 | 1 | 0:09 | 0 | 0:00 | 1 | 0:09 | 0 | 0:00 | 0 | 0:00 | 0 | 0:00 |
| 2 | 5 | 1:10 | 3 | 0:24 | 2 | 0:45 | 1 | 0:04 | 1 | 0:04 | 0 | 0:00 | 2 | 0:43 | 1 | 0:10 | 1 | 0:32 | 2 | 0:23 | 1 | 0:10 | 1 | 0:12 | 0 | 0:00 | 0 | 0:00 | 0 | 0:00 |
| 3 | 5 | 0:45 | 5 | 0:45 | 0 | 0:00 | 3 | 0:30 | 3 | 0:30 | 0 | 0:00 | 1 | 0:04 | 1 | 0:04 | 0 | 0:00 | 1 | 0:10 | 1 | 0:10 | 0 | 0:00 | 0 | 0:00 | 0 | 0:00 | 0 | 0:00 |
| 4 | 5 | 0:42 | 3 | 0:20 | 2 | 0:21 | 2 | 0:11 | 2 | 0:11 | 0 | 0:00 | 2 | 0:08 | 1 | 0:12 | 1 | 0:12 | 1 | 0:09 | 0 | 0:00 | 1 | 0:09 | 0 | 0:00 | 0 | 0:00 | 0 | 0:00 |
| 5 | 12 | 2:07 | 5 | 0:41 | 7 | 1:26 | 3 | 0:34 | 3 | 0:34 | 0 | 0:00 | 5 | 0:44 | 2 | 0:07 | 3 | 0:36 | 1 | 0:15 | 0 | 0:00 | 1 | 0:15 | 3 | 0:34 | 0 | 0:00 | 3 | 0:34 |
| 6 | 7 | 1:02 | 5 | 0:40 | 2 | 0:26 | 2 | 0:19 | 2 | 0:19 | 0 | 0:00 | 2 | 0:20 | 1 | 0:08 | 1 | 0:12 | 3 | 0:25 | 2 | 0:11 | 1 | 0:14 | 0 | 0:00 | 0 | 0:00 | 0 | 0:00 |
| 7 | 2 | 0:18 | 1 | 0:05 | 1 | 0:12 | 1 | 0:05 | 1 | 0:05 | 0 | 0:00 | 0 | 0:00 | 0 | 0:00 | 0 | 0:00 | 1 | 0:12 | 0 | 0:00 | 1 | 0:12 | 0 | 0:00 | 0 | 0:00 | 0 | 0:00 |
| 8 | 3 | 0:31 | 2 | 0:15 | 1 | 0:16 | 2 | 0:15 | 2 | 0:15 | 0 | 0:00 | 1 | 0:16 | 0 | 0:00 | 1 | 0:16 | 0 | 0:00 | 0 | 0:00 | 0 | 0:00 | 0 | 0:00 | 0 | 0:00 | 0 | 0:00 |
| 9 | 10 | 3:11 | 7 | 2:33 | 3 | 0:38 | 4 | 1:09 | 4 | 1:09 | 0 | 0:00 | 3 | 1:21 | 1 | 0:57 | 2 | 0:24 | 0 | 0:00 | 0 | 0:00 | 0 | 0:00 | 3 | 0:41 | 2 | 0:27 | 1 | 0:14 |
| 10 | 4 | 0:48 | 2 | 0:12 | 2 | 0:36 | 1 | 0:05 | 1 | 0:05 | 0 | 0:00 | 2 | 0:23 | 1 | 0:06 | 1 | 0:17 | 1 | 0:18 | 0 | 0:00 | 1 | 0:18 | 0 | 0:00 | 0 | 0:00 | 0 | 0:00 |
| 11 | 4 | 0:39 | 2 | 0:12 | 2 | 0:27 | 2 | 0:12 | 2 | 0:12 | 0 | 0:00 | 1 | 0:16 | 0 | 0:00 | 1 | 0:16 | 1 | 0:10 | 0 | 0:00 | 1 | 0:10 | 0 | 0:00 | 0 | 0:00 | 0 | 0:00 |
| 12 | 7 | 0:57 | 5 | 0:27 | 2 | 0:29 | 3 | 0:18 | 3 | 0:18 | 0 | 0:00 | 3 | 0:28 | 2 | 0:09 | 1 | 0:18 | 1 | 0:10 | 0 | 0:00 | 1 | 0:10 | 0 | 0:00 | 0 | 0:00 | 0 | 0:00 |
| 13 | 1 | 0:03 | 1 | 0:03 | 0 | 0:00 | 1 | 0:03 | 1 | 0:03 | 0 | 0:00 | 0 | 0:00 | 0 | 0:00 | 0 | 0:00 | 0 | 0:00 | 0 | 0:00 | 0 | 0:00 | 0 | 0:00 | 0 | 0:00 | 0 | 0:00 |
| 14 | 5 | 0:22 | 2 | 0:04 | 3 | 0:22 | 1 | 0:03 | 1 | 0:03 | 0 | 0:00 | 0 | 0:00 | 0 | 0:00 | 0 | 0:00 | 1 | 0:09 | 0 | 0:00 | 1 | 0:09 | 3 | 0:10 | 1 | 0:01 | 2 | 0:08 |
|  |  |  |  |  |  |  |  |  |  |  |  |  |  |  |  |  |  |  |  |  |  |  |  |  |  |  |  |  |  |  |
| **Tot** | 73 | 13:02 | 45 | 6:58 | 28 | 6:12 | 28 | 4:07 | 28 | 4:07 | 0 | 0:00 | 22 | 4:48 | 10 | 1:56 | 12 | 3:07 | 14 | 2:35 | 4 | 0:32 | 10 | 2:03 | 9 | 1:25 | 3 | 0:28 | 6 | 0:57 |
| **Mean** | 5,21 | 0:55 | 3,21 | 0:29 | 2,00 | 0:26 | 2 | 0:17 | 2,00 | 0:17 | 0,00 | 0:00 | 1,57 | 0:20 | 0,71 | 0:08 | 0,86 | 0:13 | 1,00 | 0:11 | 0,29 | 0:02 | 0,71 | 0:08 | 0,64 | 0:06 | 0,21 | 0:02 | 0,43 | 0:04 |
| **Std** | 2,99 | 0:49 | 1,85 | 0:38 | 1,71 | 0:21 | 0,96 | 0:17 | 0,96 | 0:17 | 0,00 | 0:00 | 1,45 | 0:23 | 0,73 | 0:14 | 0,86 | 0:12 | 0,78 | 0:07 | 0,61 | 0:04 | 0,47 | 0:06 | 1,28 | 0:13 | 0,58 | 0:07 | 0,94 | 0:09 |

#P = patient code, #S = sessions, UT = usage time [hh:mm], NI = non-interactive, I = interactive

**Supplementary Table 3 –** Results from the statistical analysis on the impact of non-interactive and interactive content on the differences (Post-Pre) of the ESAS items

| **(Post-Pre) ESAS item** | **Non-interactive (S.D. error)** | **Interactive (S.D. error)** |  | **p value** |
| --- | --- | --- | --- | --- |
| Pain | 0.35 (0.14) | 0.51 (0.21) |  | 0.42 |
| Tiredness | -0.4 (0.3) | 0.28 (0.48) |  | 0.15 |
| Nausea | -0.02 (0.11) | 0.08 (0.13) |  | 0.43 |
| Depression | -0.67 (0.22) | -0.30 (0.22) |  | 0.09 |
| Anxiety | -1.13 (0.24) | -0.84 (0.3) |  | 0.33 |
| Drowsiness | -0.3 (0.20) | -0.35 (0.0.31) |  | 0.85 |
| Appetite | -0.53 (0.25) | 0.18 (0.41) |  | 0.08 |
| Well-being | -1.22 (0.28) | -0.71(0.45) |  | 0.25 |
| Short of breath | -0.8 (0.25) | -0.61 (0.41) |  | 0.63 |


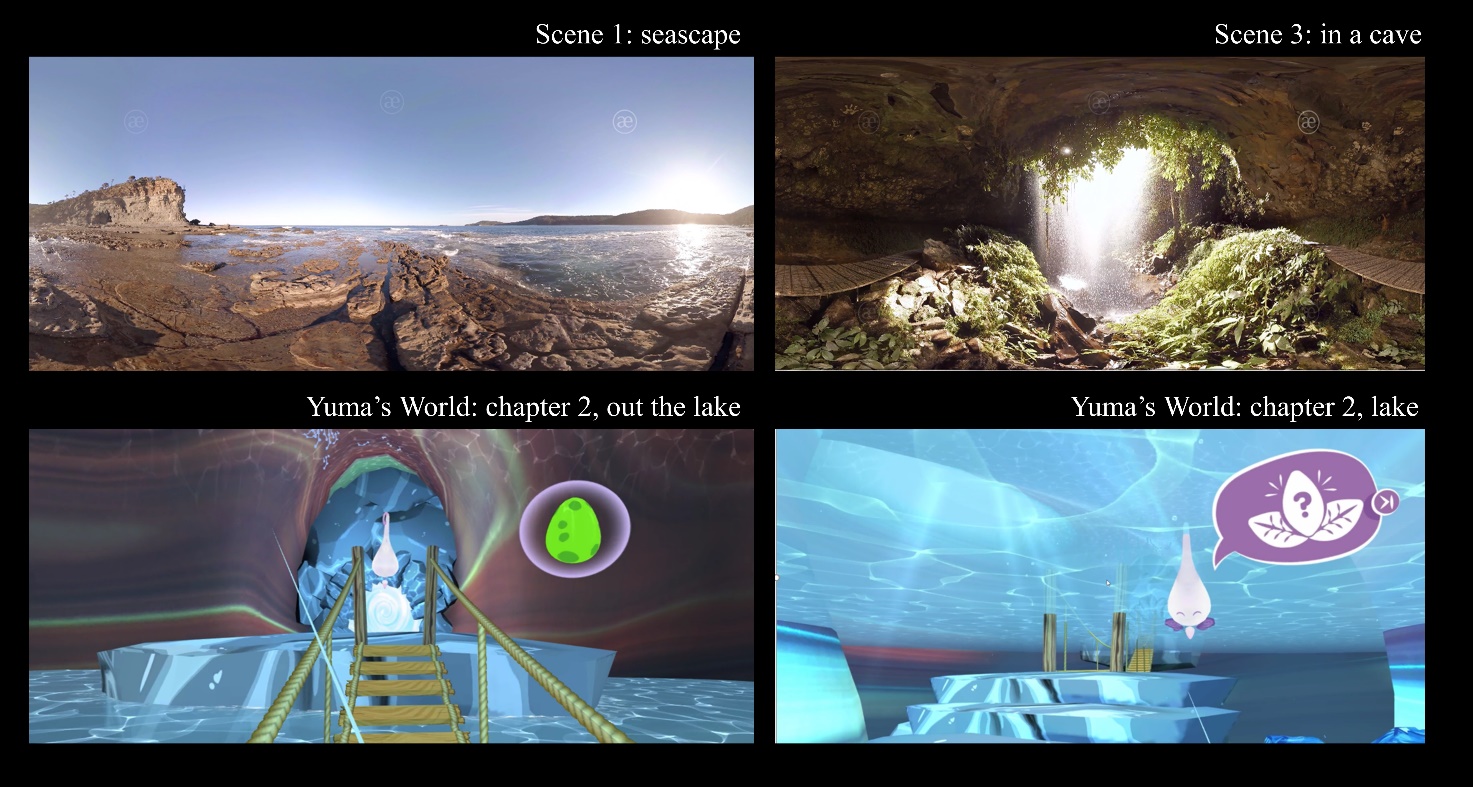


**Supplementary Figure 1** - Upper panel: examples of non-interactive contents; Lower panel: examples of interactive content specifically developed for ANT patients


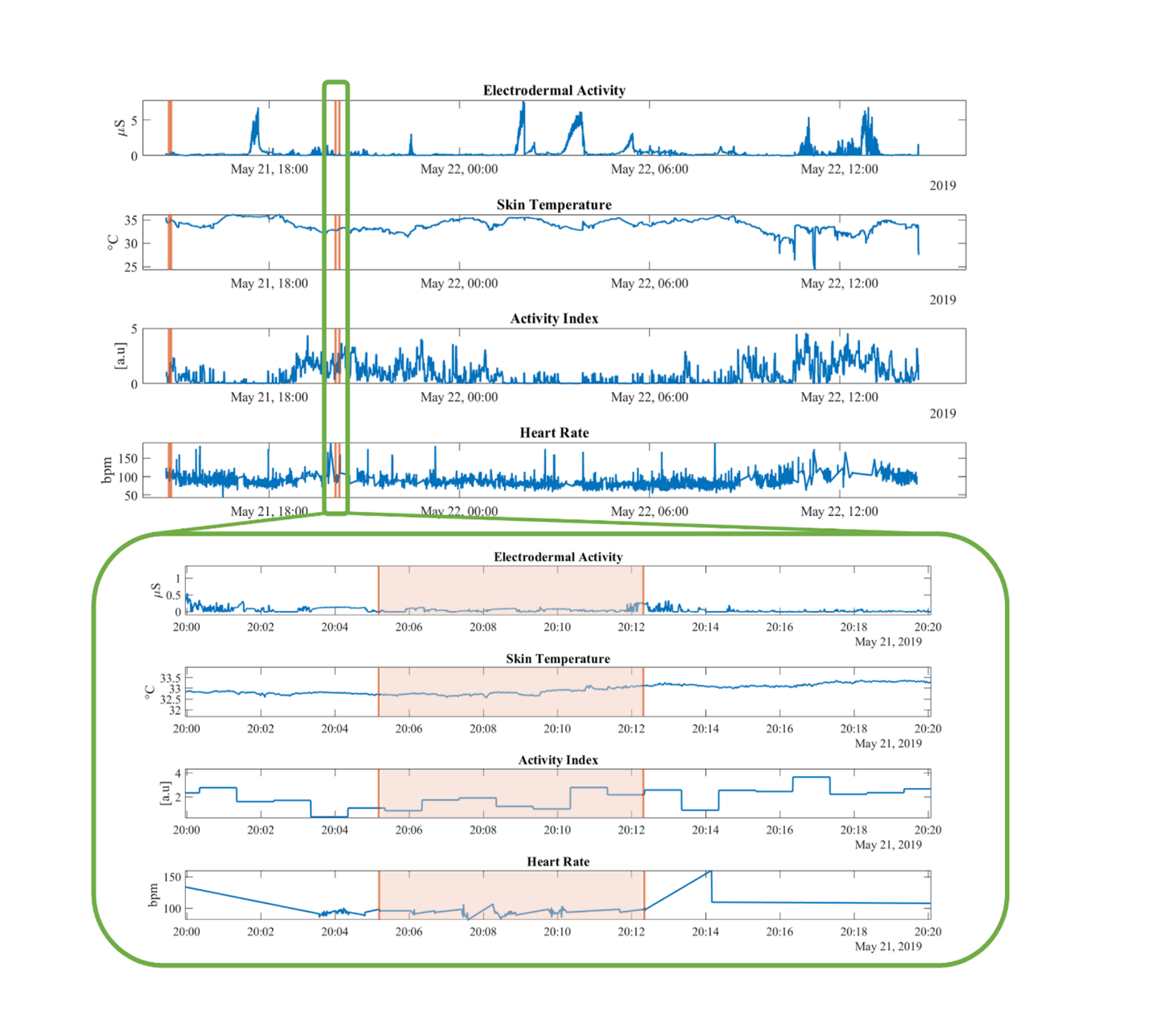


**Supplementary Figure 2** – Upper panel: example of physiological recordings (blue lines), highlighting the VR sessions (orange lines)**.** Lower pane: zoom-in of a single VR session (orange-shaded window, first orange line: session’s start, last orange line: session’s end). NB: The quick rise of the Heart Rate is likely to be due to a motion artifact
